# Bone Mineral Density and Intermuscular Fat Derived from Computed Tomography Images Using Artificial Intelligence Are Associated with Fracture Healing

**DOI:** 10.3390/bioengineering12070785

**Published:** 2025-07-19

**Authors:** Yilin Tang, Xiaodong Wang, Ming Li, Liang Jin

**Affiliations:** 1 Radiology Department, Huadong Hospital, Fudan University, Shanghai 200040, China; tyl17373562576@163.com; 2 Radiology Department, State Key Laboratory of Oncology in South China, Collaborative Innovation Center for Cancer Medicine, Sun Yat-sen University Cancer Center, Guangzhou 510060, China; 3 Shanghai Changfeng Community Health Service Center, Shanghai 200062, China; wangxiaodongting@163.com; 4 Radiology Department, Huashan Hospital Affiliated with Fudan University, Shanghai 200040, China

**Keywords:** bone mineral density, artificial intelligence, fracture, sarcopenia, computed tomography

## Abstract

**Objectives:** To employ artificial intelligence (AI) to automatically measure bone mineral density (BMD) and intramuscular fat in computed tomography (CT) images of patients with fractures and explore the association between these parameters and fracture healing. **Methods:** This retrospective study included patients who underwent baseline CT scans for rib fracture diagnosis and follow-up CT scans for fracture healing assessment at our hospital between 2012 and 2023. The volumetric BMD of the entire first lumbar vertebra (L1) and the paraspinal intramuscular fat area (PIFA) at the midsection of L1 in the baseline CT were extracted using AI. The primary outcomes, including callus formation, volume increase, and poor healing, and logistic regression were used to analyze the relationships between BMD and PIFA with primary outcomes. **Results:** Overall, 297 fractures from 53 patients (24 males; mean age: 53.83 ± 10.86 years) were included in this study. In multivariate regression analysis, a 1 standard deviation (SD) decrease in BMD was identified as an independent prognostic factor for reduced callus formation (odds ratio [OR] = 0.70, 95% confidence interval [CI] = 0.50–0.97), diminished volume increase (OR = 0.70, 95% CI = 0.51–0.96), and elevated poor fracture healing at follow-up (OR = 2.08, 95% CI = 1.38–3.13). Similarly, a 1 SD increase in PIFA was an independent prognostic factor for reduced callus formation (OR = 0.24, 95% CI = 0.16–0.37), diminished volume increase (OR = 0.33, 95% CI = 0.23–0.49), and elevated poor fracture healing at follow-up (OR = 2.09, 95% CI = 1.50–2.93). Therefore, a model combining BMD, PIFA, and clinical characteristics significantly outperformed a model that included only clinical characteristics in predicting callus formation, volume increase, and poor fracture healing, with areas under the curve of 0.790, 0.749, and 0.701, respectively (all *p* < 0.001). **Conclusions:** BMD and PIFA can be used as early predictors of fracture healing outcomes and can help clinicians select appropriate interventions to prevent poor healing.

## 1. Introduction

Bone mineral density (BMD), a quantitative measure of mineral content within bone tissues, is a critical biomarker for bone strength and structural integrity [1,2]. In 2008, the American College of Radiology revised its guidelines for assessing BMD using quantitative computed tomography (QCT), defining thresholds for osteopenia (80–120 mg/cm^3^), osteoporosis (<80 mg/cm^3^), and normal BMD (>120 mg/cm^3^) based on lumbar spine measurements [3]. Osteopenia and osteoporosis are age-related skeletal disorders characterized by diminished bone mass, compromised structural integrity, and increased fragility, which contribute to a reduced capacity for load-bearing and resistance to mechanical stress [4]. Sarcopenia, a progressive loss of muscle mass and function, is another age-related musculoskeletal condition resulting from an imbalance between muscle protein synthesis and degradation, leading to intermuscular and intramuscular adipose tissue infiltration [5,6]. Notably, the shift in global population demographics towards older age groups is expected to significantly increase the burden of sarcopenia and decreased BMD on public health.

Nonunion and delayed union of rib fractures can result in chronic pain, functional impairment, and thoracic deformities [7]. Moreover, there is often a significant delay before patients receive a definitive diagnosis of nonunion, which worsens clinical outcomes [8]. Therefore, early detection of rib fractures with a high risk of nonunion is crucial for mitigating patient morbidity and optimizing therapeutic interventions. Prior studies have demonstrated that reduced BMD and sarcopenia, as assessed through imaging modalities, are associated with increased fracture susceptibility and impaired healing [9,10,11,12,13,14,15,16,17]. Dual-energy X-ray absorptiometry (DXA) remains the gold standard for the assessment of BMD and sarcopenia [18]. However, its availability is limited in some healthcare settings, and it may not be feasible for patients with acute rib fractures or concurrent injuries [19]. Additionally, artifacts from degenerative changes, bowel contents, and vascular calcifications can confound the accuracy of DXA measurements [20]. Computed tomography (CT) is the preferred modality for diagnosing and monitoring rib fractures due to its superior resolution and ability to generate high-quality multiplanar reconstructions [21]. Additionally, CT scans performed for diagnostic purposes often yield ancillary biological data, which ensure the opportunistic evaluation of body composition without additional radiation exposure or patient time [22,23,24].

Advances in artificial intelligence (AI), particularly in deep learning, have facilitated the automated extraction and analysis of these data, enhancing their clinical applicability. Methods based on convolutional neural networks, especially the U-Net architecture, have been extensively employed in the automated measurement of body composition parameters—such as muscle and fat area/volume—in medical imaging, demonstrating reliable accuracy and practicality [25,26,27,28,29]. Meanwhile, the potential value of automatically extracted body composition parameters from medical imaging in disease diagnosis, prognosis prediction, and treatment response assessment has been increasingly recognized and validated in recent studies [30,31,32,33,34]. A recent study by Ziegelmayer et al. [32] showed that automatically quantified muscle mass and intermuscular fat from magnetic resonance imaging were significantly associated with chronic pain. Yi et al. [31] found that deep learning-based AI techniques could automatically extract BMD and intermuscular fat volume from CT images, which were negatively and positively correlated, respectively, with all-cause mortality. Despite these advances, no study has employed AI to simultaneously quantify BMD and the paraspinal intermuscular fat area (PIFA) from opportunistic CT scans to explore their association with fracture healing outcomes.

Therefore, we applied AI technology to automatically quantify the first lumbar vertebra (L1) BMD and PIFA in baseline CT scans obtained for rib fracture assessment. This study aimed to evaluate whether AI-measured BMD and PIFA on CT images are valuable early predictors of fracture healing outcomes to guide clinical decision-making and optimize intervention strategies to reduce the risk of poor healing.

## 2. Methods

### 2.1. Study Design and Data Selection

Rib fracture imaging at approximately 3 weeks post-fracture is considered indicative of a fresh fracture, which subsequently enters the reparative phase of fracture healing, characterized by blurred fracture lines and callus formation [35]. Typically, rib fractures complete the reparative phase at approximately 3 months post-fracture and then enter the remodeling phase, during which the callus is remodeled to restore bone structure without further callus formation [36]. Therefore, to investigate the effects of BMD and PIFA, patients with fresh rib fractures who underwent a baseline scan within 1 month post-injury and two CT scans within 4 months after the fracture were included in this study.

**Figure 1:**
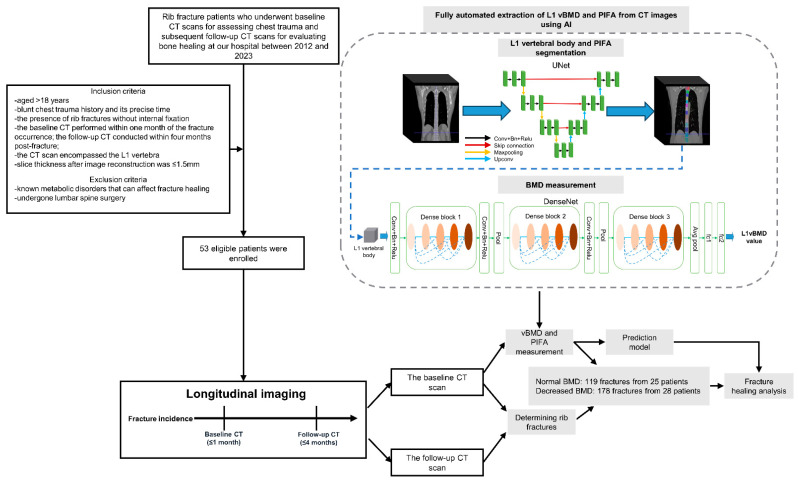
Study workflow. The UNet architecture employed in this study follows a classic encoder–decoder structure enhanced with skip connections to enable the integration of contextual and spatial information across stages. The encoder path utilizes two consecutive 3 × 3× 3 convolutional layers followed by a 2 × 2 × 2 max pooling operation (stride = 2), repeated over four levels to progressively capture features at different spatial scales. In the decoder path, feature maps are upsampled using a 2 × 2 × 2 transposed convolution layer and refined through two successive 3 × 3× 3 convolutions, also repeated across four stages. The final output is produced using a 1 × 1 convolution layer to map the feature maps to the required number of output classes. For BMD measurement, a DenseNet-based model was implemented, consisting of a feature extractor and a regression module. The extractor includes three Dense Blocks, each comprising an equal number of 1 × 1 × 1 and 3 × 3× 3 convolution layers for multiscale feature learning. The regression component applies two successive 2 × 2 × 2 global average pooling layers, with output trained to match BMD values derived from QCT scans. Input to the network is standardized to a shape of 16 × 32 × 32 voxels. Abbreviations: QCT, quantitative computed tomography; BMD, bone mineral density; Conv, convolution; BN, batch normalization; ReLU, rectified linear unit.

Retrospective unenhanced CT images were retrieved from our institution’s picture archiving and communication system for patients who presented with thoracic trauma between 2012 and 2023. The inclusion criteria included age ≥18 years, documented blunt thoracic trauma, rib fractures managed non-operatively, baseline CT obtained within 1 month of injury, follow-up CT within 4 months post-fracture, and post-reconstruction slice thickness ≤ 1.5 mm. The exclusion criteria were having undergone lumbar spine surgery and known metabolic or endocrine disorders that can impact fracture healing. Figure 1 details the patient selection process.

### 2.2. CT Acquisition Protocols

All individuals underwent scanning on two distinct CT scanners within the same hospital: (I) Revolution CT, GE Healthcare (Chicago, IL, USA); (II) Somatom Force, Siemens Healthcare (Forchheim, Germany). Non-enhanced chest CT images were obtained during a breath-hold at full inspiration. Furthermore, to ensure the quality and performance of the CT scanners over long-term use, QA phantom data were acquired monthly by separately scanning an asynchronous phantom (Mindways Software, Inc., Austin, TX, USA) using the same parameters. Imaging protocols included tube voltages of 100–120 kVp, tailored to the patient’s body habitus, with tube currents ranging from 100 to 200 mAs, pitch values of 0.75–1.50, and collimation widths of 1.00–1.25 mm. Notably, all imaging datasets were reconstructed into 512 × 512 matrices using bone or medium-resolution algorithms, with slice thickness ranging from 0.625 to 1.500 mm. The scan field encompassed the entire thoracic cage and the L1 vertebra.

### 2.3. BMD and PIFA Evaluated Using AI

The L1 vertebra is recognized as the optimal anatomical site for opportunistic BMD quantification using CT, with single-slice fat area measurements exhibiting a strong correlation with total body adiposity [37,38]. Accordingly, the L1 vertebra was selected for the quantification of volumetric BMD (vBMD) and the intermuscular fat area of the psoas, erector spinae, and multifidus muscles at its midsection. A deep learning-based, commercially available AI software (Quantitative CT-Assisted Diagnosis System, version 1.0, Huiying Medicine Technology, China) was used for the fully automatic measurements of vBMD and PIFA. This software has been approved for clinical use by the National Medical Products Administration (NMPA) of China, with a medical device registration certificate (No. 20222210119, verifiable on the NMPA official website). Its performance has been validated in two recent large-scale studies, which reported average Dice similarity coefficients greater than 0.95 between automated vertebral segmentation and manual annotations by radiologists, as well as mean differences between AI-derived and QCT-based vBMD measurements of (−0.28–2.37) and (1.30–1.92) mg/cm^3^, respectively [39,40]. Figure 1 shows a detailed visualization of the automatic AI architecture. Specifically, a three-dimensional UNet architecture facilitated the segmentation of the vertebral body, focusing on the trabecular bone while excluding the cortical shell. Subsequently, a DenseNet architecture was used to quantify vBMD, ultimately calculating BMD values in mg/cm^3^. The UNet architecture consistent with that used for vertebral segmentation was applied to extract the PIFA at the midsection of the L1 vertebra, with the input images modified to two-dimensional (Figure 1).

### 2.4. Image Analysis

Thin-slice unenhanced CT images in Digital Imaging and Communications in Medicine (DICOM) format underwent conversion to Neuroimaging Informatics Technology Initiative format for further analysis. Preprocessing steps included rescaling images to 0.7 × 0.7 × 0.7 mm voxels using linear interpolation, followed by intensity normalization (window width: 1500 Hounsfield units [HU]; window level: 500 HU).

A junior radiologist with 6 years of experience in thoracic imaging confirmed the presence of callus at all fracture sites based on diagnostic CT reports. Callus formation on follow-up CT was defined by the presence of new bony tissue at the fracture site. Callus regions were manually delineated as volumes of interest (VOIs) on the preprocessed CT images using ITK-SNAP software (version 3.8.0; www.itksnap.org). All VOIs were reviewed and the final delineations validated by a senior radiologist with over 20 years of thoracic imaging expertise.

The VOI volume was quantified using the “MeshVolume” radiomic feature in PyRadiomics, which was implemented in Python (version 3.8). This value represents the three-dimensional mesh volume generated from the VOI, calculated based on the triangulated surface model derived from the segmented region [41]. It provides an accurate representation of the actual physical space occupied by the region, typically expressed in cubic millimeters (mm^3^). Furthermore, to quantify callus volumetric changes, we calculated the percentage volume increase (PVI) for each fracture by matching callus volumes obtained from baseline and follow-up CT scans.

The clinical definitions of nonunion and delayed union varied significantly, ranging from 1 to 8 months and 2 to 12 months, respectively [42]. The U.S. Food and Drug Administration defines nonunion as a fracture that remains unhealed after 9 months, without radiographic evidence of healing in the preceding 3 months [43]. However, delayed union refers to fractures that have not achieved complete healing within 6 months. Consequently, waiting 6–9 months for definitive confirmation of poor healing may cause significant psychological distress in patients and result in missed opportunities for the timely window for intervention. The radiological signs of fracture healing include blurred fracture line margins with callus formation or mature callus after bone remodeling accompanied by the disappearance of the fracture line [35]. Therefore, we defined poor fracture healing as a fracture lacking radiographic signs of healing characterized by sharp fracture lines without periosteal reaction or callus formation on baseline or follow-up CT.

### 2.5. Statistical Analyses

All statistical analyses were conducted using R Studio ver. 4.3.1 (RStudio Team [2020]. RStudio: Integrated Development for R. RStudio, PBC, Boston, MA, USA. URL: http://www.rstudio.com/, accessed on 7 June 2023). Continuous variables were expressed as mean ± standard deviation (SD) for normally distributed data or as median and interquartile range (IQR) for non-normally distributed data. Categorical data were presented as frequencies and percentages (*n*%). Furthermore, group comparisons were performed using the Kruskal–Wallis test or t-test for continuous variables and Pearson’s chi-square test or Fisher’s exact test for categorical variables. PVI in callus volume (unit:mm^3^) between the baseline and follow-up CT examinations was calculated as follows: Volume CT2 − Volume CT1/Volume CT1. Increased PVI was defined as a >25% volume change over the interval.

Patients were initially categorized into three groups: osteopenia, osteoporosis, and normal BMD (Table S1). Therefore, to balance the sample sizes across groups, patients with osteopenia and osteoporosis were combined into a single group, referred to as the decreased BMD group. Three types of analyses were performed: (1) unadjusted, (2) adjusted for age (per decade) and sex, and (3) further adjusted for BMD and PIFA in addition to age and sex. The associations between BMD, PIFA, and outcomes such as callus formation, callus growth, and poor fracture healing were evaluated using binary logistic regression models.

Models combining sex, age, BMD, and PIFA were developed to predict callus formation, callus PVI, and poor fracture healing. These models were compared with clinical models that included only age and sex to evaluate the predictive value of BMD and PIFA. Five-fold cross-validation was used to assess the predictive accuracy of the model for fracture outcomes, and model performance was evaluated based on the area under the receiver-operating characteristic (ROC) curve [44]. The Youden index evaluates the difference between sensitivity and specificity across all potential cut-off values to identify the optimal threshold [45,46]. Therefore, in this study, we applied the Youden index to determine the optimal cut-off point for each model. The DeLong test was used to compare differences between ROC curves, with statistical significance set at *p* < 0.05 [47].

## 3. Results

### 3.1. Patient and Fracture Characteristics

Overall, 53 eligible patients were included in this study. The mean age was 53.64 ± 10.64 years, with 29 males (54.7%) and 24 females (45.3%). The average BMD was 120.45 ± 37.63 mg/cm^3^, and the average PIFA was 356.25 ± 186.12 mm^2^. Among these patients, there were 297 total fractures. The mean interval between the baseline CT scan and the fracture occurrence was 0.43 ± 0.35 months, with a follow-up CT scan performed at 2.11 ± 0.96 months post-fracture. However, the average interval between the two CT scans was 1.69 ± 0.86 months.

The normal BMD group comprised 25 patients (119 fractures), and the decreased BMD group included 28 patients (178 fractures). Notably, no statistically significant differences were observed between the normal and decreased BMD groups concerning the timing of the baseline CT (0.41 ± 0.33 vs. 0.45 ± 0.37 months, *p* = 0.378), follow-up CT (2.20 ± 0.80 vs. 2.05 ± 1.05 months, *p* = 0.191), or the interval between scans (1.79 ± 0.81 vs. 1.61 ± 0.89 months, *p* = 0.079). Additionally, the number of rib fractures (1–3, 4–7, and 8–12 ribs) or the types of fractures identified did not differ between the two groups. Table 1 presents the clinical characteristics of patients and fractures according to BMD status.

### 3.2. Associations of BMD and PIFA with Callus Formation

Decreased BMD and increased PIFA were not associated with callus formation in baseline CT scans. In the baseline CT images, callus formation was observed in 123 of 297 fractures (41.4%). Specifically, 55 of 119 (46.2%) and 68 of 178 (38.2%) fractures in the normal and decreased BMD groups, respectively, showed callus formation, with no statistically significant differences between the groups (*p* = 0.169). In the unadjusted analysis, each 1 SD decrease in BMD was significantly negatively associated with callus formation in baseline CT scans (odds ratio [OR] = 0.73; 95% CI: 0.58–0.93; *p* = 0.009). However, after adjusting for sex, age, and PIFA, the association was no longer significant (*p* > 0.05). Additionally, each 1 SD increase in PIFA did not significantly influence callus formation in baseline CT scans, regardless of all adjustments (all *p* > 0.05) (Table 2).

Furthermore, decreased BMD and increased PIFA were associated with lower rates of callus formation in follow-up CT scans. Callus formation was observed in 193 of 297 fractures (65.0%) in the follow-up CT scans, with 85 of 119 (71.4%) and 108 of 178 fractures (60.7%) in the normal and decreased BMD groups, respectively. After adjusting for age and sex, decreased BMD was significantly associated with a lower rate of callus formation (OR = 0.41; 95% CI: 0.22–0.75; *p* = 0.004). The unadjusted analysis showed that each 1 SD decrease in BMD was not significantly associated with callus formation (*p* = 0.108). However, after adjusting for age and sex, the association became significant (OR = 0.66; 95% CI: 0.49–0.89; *p* = 0.007). Therefore, adjusting for PIFA did not diminish the strength of the association, suggesting that BMD and PIFA are independent predictors. Furthermore, each 1 SD increase in PIFA was associated with a lower rate of callus formation, with an unadjusted OR of 0.32 (95% CI: 0.22–0.45; *p* < 0.001) and an adjusted OR of 0.25 (95% CI: 0.16–0.38; *p* < 0.001). The stability of the association after adjusting for BMD further supports the independence of BMD and PIFA as predictors (Table 2).

### 3.3. Associations of BMD and PIFA with Callus Volume Increase

The median PVI of the callus between baseline and follow-up CT images was 25.6% (IQR: −0.41–100%), with 148 of 297 fractures (49.8%) showing an increase in PVI.

Decreased BMD and increased PIFA were both associated with reduced callus volume. In the normal BMD group, 67 fractures (56.1%) showed callus PVI, compared with 81 fractures (45.5%) in the decreased BMD group. In the unadjusted analysis, decreased BMD was not significantly associated with callus PVI (OR = 0.65; 95% CI: 0.41–1.03; *p* = 0.069). However, after adjusting for age and sex, decreased BMD was significantly associated with reduced callus PVI (OR = 0.26; 95% CI: 0.16–0.49; *p* < 0.001). The unadjusted analysis showed that a 1 SD decrease in BMD was not significantly associated with callus PVI (*p* = 0.349); however, the association became significant after adjusting for age and sex (OR = 0.66; 95% CI: 0.49–0.89; *p* = 0.007). Consequently, adjusting for PIFA did not weaken this association, indicating that BMD and PIFA are independent predictors. Each 1 SD increase in PIFA was associated with a significant reduction in callus PVI, both unadjusted (OR = 0.32; 95% CI: 0.22–0.45; *p* < 0.001) and adjusted (OR = 0.25; 95% CI: 0.16–0.38; *p* < 0.001) for age and sex; adjusting for BMD did not weaken the association, further supporting the notion that BMD and PIFA can independently predict callus PVI (Table 2).

### 3.4. Associations of BMD and PIFA with Poor Fracture Healing

Notably, decreased BMD and increased PIFA were associated with higher rates of poor fracture healing. Among the 297 fractures, 48 cases (16.2%) exhibited poor healing. In the normal BMD group, 10 of 119 fractures (8.3%) had poor healing, compared with 38 of 178 fractures (21.3%) in the decreased BMD group, a significantly higher proportion (*p* = 0.003). Furthermore, compared with normal BMD, decreased BMD was associated with a higher likelihood of poor fracture healing, both unadjusted (OR = 2.96; 95% CI: 1.41–6.20) and adjusted (OR = 4.78; 95% CI: 2.07–11.06) for age and sex. Therefore, by analyzing the effect of each 1 SD decrease in BMD and each 1 SD increase in PIFA, the unadjusted odds ratios for poor fracture healing were 0.59 (95% CI: 0.40–0.85; *p* = 0.005) and 2.00 (95% CI: 1.49–2.69; *p* < 0.001), respectively. In the adjusted analysis, the ORs were 0.32 (95% CI: 0.20–0.53; *p* < 0.001) and 2.16 (95% CI: 1.59–2.94; *p* < 0.001). However, adjusting for BMD/PIFA did not reduce the strength of these associations, further supporting their roles as independent predictors of poor fracture healing (Table 2).

### 3.5. Predictive Value of BMD and PIFA for Fracture Healing Outcomes

**Figure 2:**
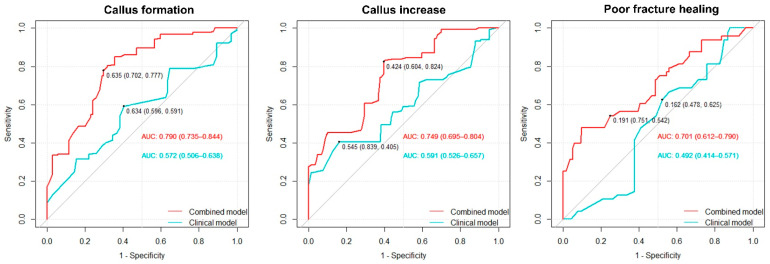
ROC curves for predicting callus formation, callus volume increase, and poor fracture healing. The coordinates marked on each ROC curve indicate the optimal cut-off value based on the Youden index, with sensitivity and specificity shown in parentheses. Abbreviations: AUC, area under the ROC curve; ROC, receiver operating characteristic.

Models combining sex, age, BMD, and PIFA were developed to predict callus formation, callus PVI, and poor fracture healing. These models were compared with clinical models that included only age and sex to evaluate the predictive value of BMD and PIFA. Model performance was evaluated using ROC curves (Figure 2).

For predicting callus formation, the combined model demonstrated superior predictive performance, with an AUC of 0.790 (95% CI: 0.735–0.844), compared with the clinical model (AUC = 0.572; 95% CI: 0.506–0.638; *p* < 0.001). Employing the optimal cut-off point of 0.635, the combined model exhibited a sensitivity of 70.2% and a specificity of 77.8%. In comparison, the clinical model, using the optimal cut-off point of 0.634, yielded a sensitivity of 59.6% and a specificity of 59.1%.

Similarly, for predicting callus PVI, the combined model had an AUC of 0.749 (95% CI: 0.695–0.804), significantly outperforming the clinical model, which had an AUC of 0.591 (95% CI: 0.526–0.657; *p* < 0.001). Employing the optimal cut-off point of 0.424, the combined model exhibited a sensitivity of 60.4% and a specificity of 82.4%. In comparison, the clinical model, using the optimal cut-off point of 0.545, yielded a sensitivity of 83.9% and a specificity of 40.5%.

For predicting poor fracture healing, the combined model achieved an AUC of 0.701 (95% CI: 0.612–0.790), whereas the clinical model exhibited poor predictive ability with an AUC of 0.492; 95% CI: 0.414–0.571. However, the inclusion of BMD and PIFA significantly enhanced the performance of the predictive models across all outcomes (*p* < 0.001). At optimal cut-off points of 0.191 for the combined model and 0.162 for the clinical model, the former exhibited 70.2% sensitivity and 77.8% specificity, whereas the latter demonstrated 59.6% sensitivity and 59.1% specificity.

## 4. Discussion

In this study, we employed AI to automatically extract L1 BMD and PIFA from opportunistic CT images initially acquired for rib fracture assessment. We found that while baseline BMD and PIFA did not correlate with callus formation in initial CT scans, they were significantly associated with callus formation, callus volume increase, and poor fracture healing in follow-up scans, independent of sex and age. Furthermore, models integrating BMD, PIFA, and clinical characteristics demonstrated superior predictive accuracy for fracture healing outcomes compared with those relying solely on clinical variables. These findings suggest that BMD and PIFA are independent predictors of fracture healing and serve as valuable prognostic markers.

Fracture healing is a multifaceted process influenced by biomechanical, molecular, and cellular factors. Reduced BMD compromises this process by impairing bone biomechanical properties, diminishing new bone formation, and increasing osteoclast activity while reducing osteoblast function and altering inflammatory responses [48,49,50]. Previous studies have extensively documented the adverse effects of decreased BMD on fracture healing [51,52]. At the molecular level, dysregulation in the secretion of osteokines such as RANKL and sclerostin by bone tissue has been implicated in impaired fracture healing under conditions of low BMD. However, clinical evidence regarding the impact of BMD reduction on fracture healing remains inconsistent. A retrospective study of 206 vertebral fracture patients demonstrated, through quantitative analyses of bone morphology at different healing stages (e.g., woven bone volume/structure volume), that osteoporosis significantly delayed fracture healing by suppressing osteogenic capacity after adjusting for age and sex [52]. These findings are consistent with the results of our study. In contrast, a retrospective study of 461 upper limb fracture patients found no significant association between osteoporosis and delayed healing [53]. Unadjusted confounders (e.g., age/sex) and limited poor-healing cases (11/461) may underlie its low statistical power. To date, no studies have explored this issue using AI-derived BMD from opportunistic CT imaging.

Emerging studies also emphasize the significant role of muscle in fracture healing, primarily through muscle-secreted myokines that facilitate muscle–bone crosstalk [54,55]. Li et al. [56] reported that myokine irisin facilitates fracture healing by enhancing the process of endochondral ossification. Zhang et al. [57] observed that osteoporotic mice with concurrent sarcopenia displayed notably smaller callus volumes compared with those without sarcopenia. They also found that impaired healing in sarcopenic mice was associated with elevated myostatin expression, which inversely correlated with callus formation [55]. These findings align with our results and suggest that the influence of sarcopenia on fracture healing may be independent of osteoporosis. Intramuscular fat quantification on CT, particularly in paraspinal muscles, is widely used as an indicator of muscle depletion and may provide insights into muscle’s role in fracture healing [9,58,59,60].

Moreover, osteokines and myokines secreted by bone and muscle not only regulate fracture healing but also mutually influence anabolic metabolism, affecting bone and muscle quality [61]. This bidirectional interaction frequently gives rise to a vicious cycle of bone loss and muscle depletion, which is prevalent in aging and various pathologies [62,63]. Therefore, investigating the potential synergistic effects of osteopenia and sarcopenia on fracture healing is of great significance. However, current evidence from both human clinical studies and animal experiments has not yet fully elucidated this potential mechanism, and its specific role in fracture outcomes remains to be systematically explored.

Opportunistic CT provides valuable supplementary data beyond its primary diagnostic purpose, including the assessment of asymptomatic osteoporosis, sarcopenia, and fracture prediction [64,65,66,67,68]. A notable strength of this study is the use of AI for automated data extraction from opportunistic CT images. To our knowledge, this is the first study to examine associations among bone, muscle, and fracture healing using opportunistic screening, thereby offering empirical insights from real clinical scenarios. This research enhances our understanding of the roles of BMD and PIFA in fracture healing and suggests their potential as predictive markers for early detection of healing progress.

This study has several limitations despite its strengths. First, as a retrospective observational study, it may be subject to selection bias and unmeasured confounding factors such as physical activity, smoking, dietary habits, and medication that may affect BMD and PIFA. Second, differentiating the effects of BMD and PIFA from those of natural aging on fracture healing remains challenging [69]. Third, the assessment of fracture healing was based solely on radiographic criteria, without consideration of clinical symptoms such as pain or functional impairment. However, radiographic findings do not always align with symptoms, potentially leading to over- or underestimation of healing outcomes. These limitations highlight the multifactorial and complex nature of fracture healing, underscoring the need for future studies to adopt an integrative approach that systematically incorporates physiological, pharmacological, lifestyle, and sociodemographic factors to elucidate the underlying mechanisms. Integrating radiographic evaluation, functional assessments, and patient-reported outcomes is essential for a more comprehensive and clinically relevant assessment of fracture healing. Such an approach would enhance the clinical utility of BMD and PIFA as early predictive markers and their potential application in optimizing care and guiding treatment strategies for patients with rib fractures. Fourth, the accuracy of AI-based BMD and PIFA measurements depends on precise segmentation of vertebral and adipose regions, which may be affected by vertebral degeneration, bone islands, or subcutaneous edema [29,70,71]. The AI software was mainly trained on Chinese data, which may cause ethnic bias and affect accuracy in diverse populations. Moreover, the findings are based on non-contrast CT and need validation for contrast-enhanced scans. Therefore, more diverse datasets, including variations in anatomical structures, ethnicity, and scanning parameters, will be needed to minimize potential biases and develop more robust measurement methods. Finally, the relatively small sample size and the absence of a multicenter design may have constrained data diversity, restricting the generalizability of the findings. Future prospective studies are planned to incorporate rigorous power analysis and sample size estimation during the study design phase to ensure adequate statistical power, and to expand data sources to include multicenter cohorts to validate the findings across broader and diverse populations before clinical application.

In conclusion, our study demonstrates a significant association between AI-derived BMD and PIFA from opportunistic CT scans and rib fracture healing outcomes. With advancements in measurement algorithms and the increasing availability of opportunistic imaging, musculoskeletal biomarkers extracted from CT scans may serve as a valuable supplement to existing risk assessment frameworks, offering objective support for personalized management strategies. Clinically, these indicators may assist in guiding early interventions such as timely initiation of bone- and muscle-strengthening pharmacotherapy and tailored lifestyle modifications to enhance fracture healing. In identified patients at high risk of nonunion, they may also help identify candidates for early surgical fixation. Incorporating these quantitative indicators into routine imaging workflows could facilitate more proactive and individualized management of rib fractures.

## Figures and Tables

**Table 1:** Clinical characteristics of patients and fractures.

Characteristics	Total	Normal BMD	Decreased BMD	*p*-Value
Number of patients	53	25	28	
Age (years)	53.64 ± 10.64	48.72 ± 8.18	54.95 ± 10.11	<0.001
Sex (% male)	29 (54.7%)	15 (60%)	14 (50%)	0.650
Number of fractures on the ribs	5 (3–8)	5 (3–6.5)	5.5 (2.25–10)	0.299
Rib fractures				
Number of fractures	297	119	178	
Age (years)	56.35 ± 10.96	49.45 ± 8.66	60.96 ± 9.88	<0.001
Sex (% male)	162 (54.5%)	63 (52.9%)	99 (59.9%)	0.650
Fractured site				
1–3	38 (12.8%)	10 (8.4%)	28 (15.7%)	0.064
4–7	175 (58.9%)	71 (59.7%)	104 (58.4%)	0.832
8–12	84 (28.3%)	38 (31.9%)	46 (21.4%)	0.253
Fracture type				
Displaced	104 (39.2%)	44 (37.0%)	60 (33.7%)	0.563
Non-displaced	193 (60.8%)	75 (63.0%)	118 (66.3%)	
Callus				
Baseline CT	123 (41.4%)	55 (46.2%)	68 (38.2%)	0.169
Follow-up CT	193 (65.0%)	85 (71.4%)	108 (60.7%)	0.057

For categorical variables, data within parentheses are presented as percentages; for continuous variables, characteristics are displayed as mean ± standard deviation or medians with IQR. BMD, bone mineral density.

**Table 2:** Univariate and multivariate analyses of BMD and PIFA associated with fracture healing outcomes.

Parameters	Callus in Baseline CT			Callus in Follow-Up CT			Callus Increase			Poor Fracture Healing		
	OR	95% CI	*p* Value	OR	95% CI	*p* Value	OR	95% CI	*p* Value	OR	95% CI	*p* Value
Decreased BMD												
Unadjusted	0.72	0.45–1.15	0.719	0.62	0.38–1.02	0.58	0.65	0.41–1.03	0.069	2.96	1.41–6.20	0.004
Age- and sex-adjusted	1.47	0.81–2.65	0.207	0.41	0.22–0.75	0.004	0.36	0.13–0.49	<0.001	4.78	2.07–11.06	<0.001
BMD, per 1 SD decrease												
Unadjusted	0.73	0.58–0.93	0.009	1.22	0.96–1.56	0.11	0.91	0.73–1.15	0.439	1.82	1.27–2.62	0.001
Age- and sex-adjusted	0.95	0.72–1.26	0.74	0.66	0.49–0.89	0.007	0.64	0.47–0.86	0.003	2.28	1.52–3.42	<0.001
Age-, sex-, and PIFA-adjusted	0.94	0.70–1.25	0.647	0.69	0.50–0.97	0.032	0.70	0.51–0.96	0.026	2.08	1.38–3.13	<0.001
PIFA, per 1 SD increase												
Unadjusted	0.96	0.76–1.22	0.761	0.32	0.22–0.45	<0.001	0.45	0.33–0.62	<0.001	2.00	1.49–2.69	<0.001
Age- and sex-adjusted	1.09	0.86–1.39	0.479	0.25	0.16–0.38	<0.001	0.33	0.22–0.48	<0.001	2.16	1.59–2.94	<0.001
Age-, sex-, and BMD-adjusted	1.10	0.86–1.41	0.438	0.24	0.16–0.37	<0.001	0.33	0.23–0.49	<0.001	2.09	1.50–2.93	<0.001

BMD, bone mineral density; SD, standard deviation; OR, odds ratio; PIFA, paraspinal intramuscular fat area; CI, confidence interval.

## Data Availability

Relevant datasets from this study will be made available by the corresponding author upon reasonable request.

## Supplementary Materials

The following supporting information can be downloaded at: https://www.mdpi.com/article/10.3390/bioengineering12070785/s1, Table S1: Clinical characteristics of patients.
