# Supplementary material for: Bone Mineral Density and Intermuscular Fat Derived from Computed Tomography Images Using Artificial Intelligence Are Associated with Fracture Healing
Source: Bioengineering (Basel). 2025 Jul 19;12(7):785. doi: 10.3390/bioengineering12070785 (PMC12293029; doi:10.3390/bioengineering12070785)
Supplement: Supplementary file 1 [file bioengineering-12-00785-s001.zip › bioengineering-3699130-supplementary.pdf]

Table S1. Clinical characteristics of patients.

	Total	Normal BMD (n = 25)	Osteopenia (n = 20)	Osteoporosis (n = 8)	*P*-value
Age (years)	53.64±10.64	48.72±8.18	54.95±10.11	65.75±8.70	<0.001
Sex (% male)	29 (54.7%)	15 (60%)	10 (50%)	4 (50%)	0.766
Number of fractures on the ribs	5 (3–8)	5 (3–6.5)	5 (2.5–9.5)	8.5 (3.25–10.75)	0.231
